# Isolated REM sleep behavior disorder is associated with altered 24-h rest-activity measures

**DOI:** 10.3389/frsle.2023.1286124

**Published:** 2023-10-30

**Authors:** Joseph R. Winer, Renske Lok, Adrian Ekelmans, Flavia Bueno, Kathleen L. Poston, Jamie M. Zeitzer, Emmanuel H. During

**Affiliations:** ^1^Department of Neurology and Neurological Sciences, Stanford University, Stanford, CA, United States; ^2^Department of Psychiatry and Behavioral Sciences, Stanford University, Stanford, CA, United States; ^3^Mental Illness Research Education and Clinical Center, VA Palo Alto Health Care System, Palo Alto, CA, United States; ^4^Department of Neurology, Icahn School of Medicine at Mount Sinai, New York, NY, United States

**Keywords:** REM sleep behavior disorder, Parkinson's, dementia, actigraphy, rest-activity rhythms, prodrome

## Abstract

**Introduction:**

Isolated REM sleep behavior disorder (iRBD), the loss of motor inhibition during REM sleep, is a symptom of prodromal Lewy body disease, with over 80% of iRBD patients progressing to Parkinson's disease or dementia with Lewy bodies. Disruption of rest-activity patterns, an established predictor of Parkinson's disease, has not been well characterized in patients with iRBD. Here, we tested the hypothesis that accelerometer-based measures of 24-h activity would indicate greater fragmentation and variability in patients with iRBD relative to matched healthy controls.

**Materials and methods:**

Patients with iRBD (*N* = 38) had 24-h activity monitored for (mean ± SD) 24.6 ± 8.8 days using an Axivity wrist-worn accelerometer. Age, sex, and body mass index matched healthy older adults (*N* = 119) were selected as controls. Raw accelerometer data were processed and nonparametric and cosinor measures of 24-h activity were calculated. Functional principal component analyses (fPCA) were applied to detect differences in 24-h activity patterns.

**Results:**

Compared to matched controls, individuals with iRBD had significantly lower cosine amplitude, mesor, and activity during their most active 10 hours, reflecting overall lower levels of activity and disrupted activity. They also had significantly increased movement during the night (L5). FPCA indicated that decreased daytime and increased nighttime activity may explain overall differences observed in iRBD.

**Conclusion:**

Multiple metrics of rest-activity rhythms support the hypothesis that 24-h activity measures are altered in iRBD. This dysfunction may reflect degeneration of sleep-wake regulating circuits, representing a symptom of iRBD and indicating the early stages of Lewy body disease.

## Introduction

Isolated REM sleep behavior disorder (iRBD), the loss of motor inhibition during REM sleep (Schenck et al., 1986), is a symptom of prodromal Lewy body disease, with over 80% of iRBD patients eventually progressing to Parkinson's disease (PD) or dementia with Lewy bodies a decade from diagnosis (Galbiati et al., 2019; Postuma et al., 2019). In these patients, iRBD is often accompanied by other prodromes signaling progressing neurodegeneration, affecting olfaction, autonomic, cognitive, psychiatric domains, and motor function (Högl et al., 2018). Sleep and wake disturbances are also commonly reported in patients with iRBD, in the form of insomnia, fragmented and non-restorative sleep, reduced daytime alertness and fatigue. The pathophysiology underlying some of these changes is not fully elucidated, however it may be due to direct disruption of sleep-wake circuits by synuclein pathology and alteration of neurotransmission regulating sleep and wake, which can all result in disruption of the 24-h rest-activity rhythms (RAR), beyond what is normally observed with age. RAR have been studied in neurodegenerative diseases, including Alzheimer's and PD, in which alterations can be observed years prior to diagnosis (Tranah et al., 2011; Leng et al., 2020). Those alterations have been also recently described in iRBD (Filardi et al., 2020; Liguori et al., 2021) and could possibly indicate a higher phenoconversion rate to clinically-manifest PD or dementia with Lewy bodies (Feng et al., 2020). The evidence is however limited to three studies with generally small sample sizes and inconsistent findings from 1-week actigraphy collection. We aimed to replicate prior findings and better characterize RAR alterations by analyzing RAR fragmentation (intradaily variability, IV) and consistency (interdaily stability, IS), as well as apply functional principal component analysis (fPCA) in a well-characterized cohort of patients with iRBD compared to a large healthy control group.

## Materials and methods

### Participants

Thirty-eight adult participants with definite iRBD based on the International Classification of Sleep Disorders, third edition (ICSD-3) were recruited from the Stanford Sleep Clinic between April 2021 and March 2022. Patients with narcolepsy, overt synucleinopathy, or dementia of any type were excluded from participating in this study. All participants were requested to wear an actigraph continuously on the dominant wrist for at least 14 days and up to 28 days.

Accelerometer data for control participants were obtained from the UK Biobank accelerometer dataset (Doherty et al., 2017). The UK Biobank is a large ongoing community-based sample of adults living in England, Wales, and Scotland that does not exclude based on any health condition. Data were downloaded March 2022 (UK Biobank application #63099). UK Biobank accelerometer data were collected from June 2013 to January 2016 and included 103, 670 total individuals who were asked to wear the device on their dominant wrist for seven days. After accounting for missing and unreliable data, data collected during daylight savings time, and data with <5 days of recording, the remaining accelerometer data sample comprised 82,829 individuals. From this sample we matched 119 individuals to the iRBD sample by age, sex, and body mass index using the MatchIt package in R (Ho et al., 2007). Controls were excluded if they had a diagnosis of PD at any time during the UK Biobank study by self-report or electronic medical records.

The Stanford Sleep Clinic study was approved by the Institutional Review Board of Stanford, and approval for the UK Biobank study was obtained from the North West Multicentre Research Ethics Committee, the National Information Governance Board for Health and Social Care in England and Wales, and the Community Health Index Advisory Group in Scotland. All participants provided written informed consent.

#### Accelerometer data

All accelerometer data were collected using tri-axial accelerometry (Axivity, Newcastle upon Tyne, UK). Stanford Sleep Clinic (iRBD) data were collected using the AX6 device at either 25 Hz or 100 Hz, and UK Biobank (control) data were collected using the AX3 device at 100 Hz.

#### Preprocessing

Processing was identical for the Stanford Sleep Clinic and UK Biobank accelerometer data. Raw data (.cwa files) were down-sampled to 30 second epochs using the biobank accelerometer analysis package in Python v3.6.1 (Doherty et al., 2017). Non-wear time was defined as stationary episodes lasting for at least 60 min in which all three axes had a standard deviation of <13.0 mg. If present, non-wear segments were automatically imputed using the median of similar time-of-day vector magnitude and intensity distribution data points with 30-second granularity on different days of the measurement (Weed et al., 2022). Following these preprocessing steps, we derived the following six metrics.

#### Cosinor analyses

Cosinor analyses (fitting a cosine wave to the data) were performed using the cosinor2 package in R (Cornelissen, 2014) and resulted in two metrics of interest: (1) mesor (midline estimating statistic of rhythm, rhythm-adjusted mean activity, or mean cosine-adjusted activity) and (2) amplitude (half the difference between peak and nadir of fitted cosine wave).

#### Non-parametric analyses

Non-parametric analyses were conducted with nparACT package in R (Van Someren et al., 1999; Blume et al., 2016) to derive four metrics: (1) intradaily variability (IV; fragmentation of activity within 24-h periods), (2) interdaily stability (IS; regularity of activity across 24-h periods), (3) activity level during the least active 5 h (L5), and (4) activity level during the most active 10 h (M10). IV values can vary from 0 to 2, with higher values indicating greater fragmentation. IS values can vary from 0 to 1, with lower values indicating lower regularity. The start times of L5 and M10 were additionally extracted for comparison between groups.

#### Functional principal component analyses

Functional principal component analyses (fPCA) were performed using the fPCA package in R (Peng and Paul, 2009). Following previously established methods (Zeitzer et al., 2013) each individual's 24-h median accelerometer data was fit with a nine-Fourier-based function. These functions were examined with functional data analysis to determine orthogonal components that explained the most variance across individuals. The first four fPCA components were used for analysis. The analysis resulted in component scores for every individual, which reflect the magnitude of contribution of a given component to that individuals' 24-h activity pattern. These scores were extracted and compared between individuals with iRBD and matched controls. As the results of fPCA are dependent on the specific subsets of data analyzed, we used a 1:1 match for this analysis (*n* = 38 iRBD patients; *n* = 38 age, sex, and BMI matched controls).

### Statistical analysis

Demographic differences were tested with *t*-tests for continuous variables and χ^2^ tests for categorical variables. Differences between iRBD and matched control groups in 24-h activity metrics were assessed using Mann–Whitney U groupwise comparisons. Non-parametric tests were applied because of small group sizes and because they do not require the data to be normally distributed. Effect sizes were calculated using Cohen's d.

Since the iRBD dataset actigraphy recording time was longer than the matched controls (24.6 ± 8.8 days vs. 6.84 ± 0.4 days), and the effects of recording duration on cosinor and non-parametric measures are not well studied, we performed a sensitivity analysis that truncated the iRBD recordings to the first 7 days of recording.

## Results

**Figure 1:**
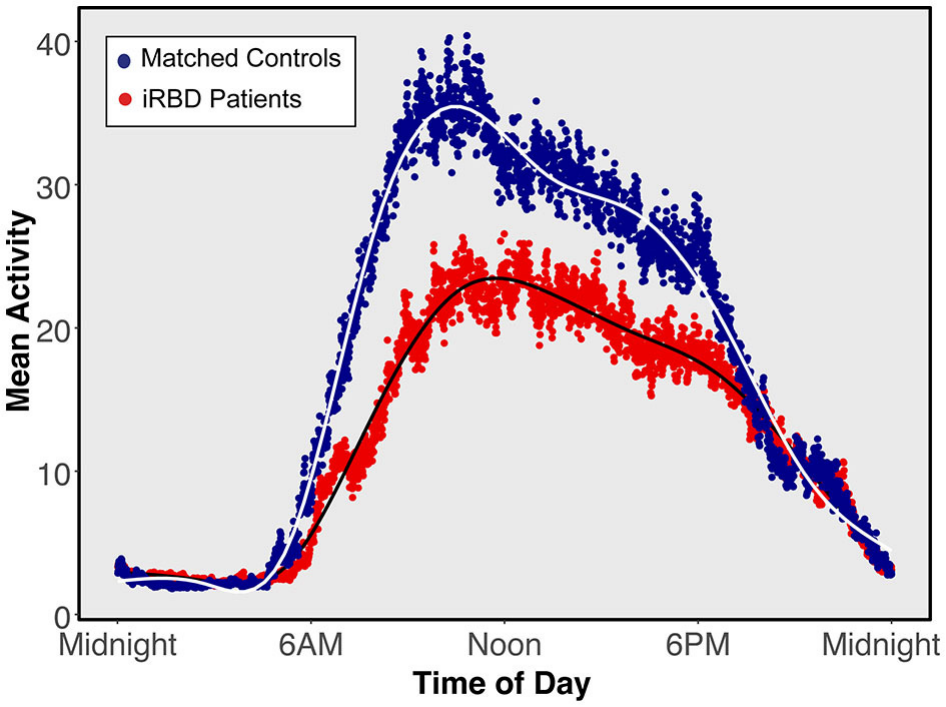
Mean 24-h activity patterns for individuals with isolated REM sleep behavior disorder and matched controls. Average activity curves in individuals with isolated REM sleep behavior disorder (red) or matched individuals (blue). Each plotted point represents a 30-s epoch of average activity.

Participant demographics showed no significant between-group differences (summarized in Table 1). The iRBD group consisted of a majority of Caucasian males with a mean age of 68.0 and 7.6 years of reported RBD symptoms. A visualization of mean 24-h activity patterns across individuals with iRBD and matched controls is presented in Figure 1.

**Table 1:** Demographics and 24-h rest-activity rhythm metrics.

	**iRBD**	**UK biobank controls**	***p*-value**
*N*	38	119	
Age	68.0 ± 6.5	66.2 ± 7.0	ns
Female, *N* (%)	7 (18)	31 (26)	ns
Caucasian, *N* (%)^*^	36 (95)	110 (99)	ns
BMI	26.3 ± 3.7	25.4 ± 3.5	ns
Years from reported RBD symptom onset	7.58 ± 3.1	n/a	-
Days recorded	24.6 ± 8.8	6.84 ± 0.4	*p < * 0.001
REM sleep without atonia index^**^	0.65 ± 0.26	n/a	-
Medication use			
Melatonin, *N* (%)	25 (66)	n/a	-
Clonazepam, *N* (%)	18 (47)	n/a	-
Rivastigmine, *N* (%)	5 (13)	n/a	-
Pramipexole, *N* (%)	2 (5)	n/a	-
Amplitude	17.0 ± 8.8	23.7 ± 8.4	*p < * 0.001
Mesor	21.2 ± 7.1	27.5 ± 7.4	*p < * 0.001
Intradaily variability	1.01 ± 0.3	0.93 ± 0.2	ns
Interdaily stability	0.53 ± 0.1	0.53 ± 0.1	ns
Least active 5 h	3.89 ± 1.5	3.11 ± 0.9	*p =* 0.001
Least active 5 h start time	0:38 ± 1:26	0:14 ± 1:19	ns
Most active 10 h	34.7 ± 13.9	47.5 ± 14.2	*p < * 0.001
Most active 10 h start time	8:45 ± 1:42	7:48 ± 1:32	*p =* 0.001

Actigraphy metrics for patients with iRBD are calculated from recordings truncated to the first 7 days, and p-values reflect these comparisons. Demographic differences were tested with t-test for continuous variables and χ^2^ tests for categorical variables. Rest-activity rhythm metric differences were tested with Mann–Whitney U groupwise comparisons.

* Ethnic background information was not available for 8 UK Biobank participants.

** REM sleep without atonia index was not available for 5 patients with iRBD.

### 24-h rest-activity patterns

**Figure 2:**
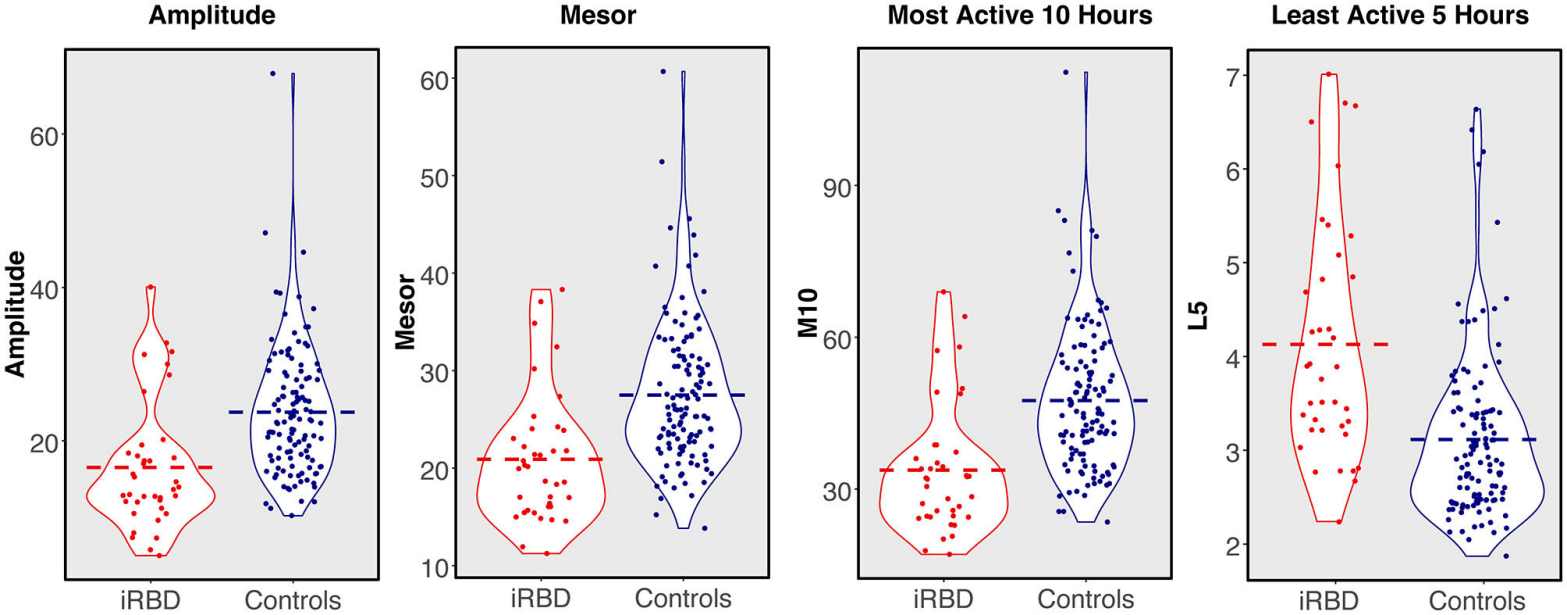
24-h rest-activity rhythm metrics in individuals with isolated REM sleep behavior disorder compared to matched controls. Dashed bar represents group mean.

Rest-activity metrics are compared in Table 1 and differences between individuals with iRBD and matched controls are visualized in Figure 2. Patients with iRBD had lower amplitude (*W* = 1056, *p* < 0.001, *d* = −0.86), mesor (*W* = 1016, *p* < 0.001, *d* = −0.91), and M10 (*W* = 925, *p* < 0.001, *d* = −0.99) relative to matched controls, reflecting overall lower levels of activity and more disrupted rhythmicity. They also had higher L5 (*W* = 3485, *p* < 0.001, *d* = 1.02) reflecting greater movement during the night. In a sensitivity analysis where iRBD recordings were truncated to the first 7 days, these comparisons remained statistically significant.

M10 start times were later in the day for iRBD patients (*W* = 3141, *p* < 0.001, *d* = 0.68), but L5 start times did not differ (*W* = 2447, *p* = 0.44, *d* = 0.17), suggesting iRBD patients may have woken up later in the morning but went to the bed around the same time relative to controls. These comparisons were similar when truncating iRBD recordings to the first 7 days.

Regularity (IS) was significantly lower in iRBD patients (*W* = 1427.5, *p* < 0.001, *d* = 0.66) but this difference was no longer present when truncating iRBD recordings to the first 7 days (*W* = 2201.5, *p* = 0.81, *d* = 0.008), suggesting that the IS measure may be biased by the duration of actigraphy recording. There was no difference between groups for fragmentation (IV, *W* = 2604, *p* = 0.16, *d* = 0.34).

### Functional principal components analysis

**Figure 3:**
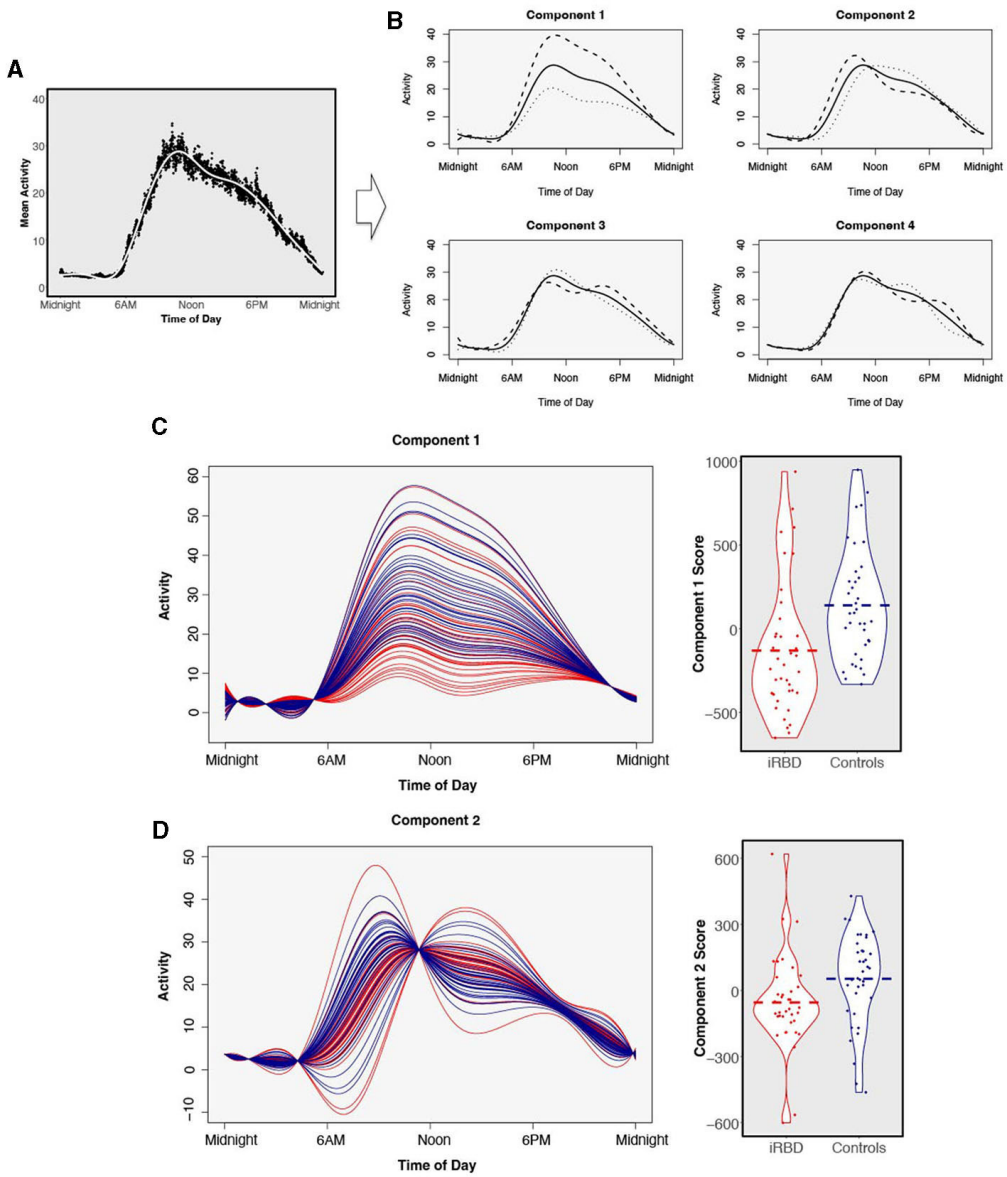
Functional principal components analysis. Four components were derived from 24-h data from *n* = 38 individuals with isolated REM sleep behavior disorder and *n* = 38 matched controls. **(A)** The average 24-h activity pattern across all individuals. **(B)** The solid line represents the same average activity curve in each of the four plots, plotted against clock time. Each of the four components is visualized by showing the average weight of positive fPCA scores (dashed) or negative scores (dotted) added to the average activity pattern. **(C)** Comparison of functional principal component scores in individuals with isolated REM sleep behavior disorder (red) and matched controls (blue) for component 1 and **(D)** component 2. Components are visualized as individual participants' curves (left) and magnitude scores (right). There is a separation of individuals with isolated REM sleep behavior disorder and matched controls. Dashed bar represents group mean.

The shapes of the components explaining the variance in 24-h activity are presented in Figure 3. Component 1 explains 62% of variance and captures daytime activity levels and inactivity in the latter half of the night. Elevated component 2 (explaining 19% of variance) represents an earlier and higher morning activity peak, while lower component 2 represents later wake and a sustained level of activity. Component 3 appears to capture the extent to which individuals had a mid-day dip in activity, explaining 7.6% of variance. Component 4, explaining 6.2% of variance, represents the timing of an afternoon peak of activity.

Comparing the magnitude scores for the four fPCA components between individuals with iRBD and matched controls revealed large differences in the first two activity components which are visualized in Figures 3C, D. Component 1 was significantly reduced in iRBD patients, suggesting both lower activity during the day and greater movement in the night (Figure 3C, *W* = 368, *p* < 0.001, *d* = 0.74). Component 2 was also reduced in iRBD, suggesting a lower level of morning activity but a sustained level of activity during the day (Figure 3D, *W* = 438, *p* = 0.005, *d* = −0.52). Component 3 was slightly elevated in iRBD patients **(***W* = 890, *p* = 0.048, *d* = 0.32). Component 4 did not differ between iRBD patients and controls (*W* = 709, *p* = 0.95, *d* = 0.06).

## Discussion

Our data indicate that individuals with iRBD, as compared to age-matched individuals without significant neurologic disorder, have reduced activity during the daytime, elevated activity at night, and notable differences in the distribution of activity levels. This study replicates and extends prior findings or RAR alterations in iRBD and provides additional insights into the specific differences in 24-h activity patterns compared to matched controls using fPCA.

We found that individuals with iRBD differed from healthy individuals across several metrics of rest-activity rhythm integrity. The iRBD patients had reduced diurnal amplitude, reduced mesor, and daytime activity (M10) that was both reduced and delayed, starting later in the morning relative to controls. They also had higher levels of nighttime activity (L5). We found 24-h activity differences with fPCA, which revealed that three orthogonal 24-h activity components were significantly different between iRBD and controls. One component capturing activity elevation during daytime and reduction in the latter part of the night and another capturing earlier and higher morning activity levels were lower in iRBD, and a third capturing the bimodal (morning and afternoon) daytime distribution of activity was elevated in iRBD. Overall, our results support that in iRBD, 24-h activity patterns are affected independently of the mere effect of age or gender, and may as such be independent clinical markers of disease state and/or disease stage.

Our results replicate previous reports that overall activity (mesor) and daytime activity (M10) are reduced in iRBD relative to healthy controls (Feng et al., 2020; Liguori et al., 2021). Like others, we found no difference in 7-day measurements of 24-h fragmentation (IV) or regularity across days (IS) when comparing iRBD patients and controls (Feng et al., 2020; Liguori et al., 2021). We observed lower diurnal amplitude in iRBD, which did not significantly differ in the study by Feng and colleagues but was shown to predict 2-year phenoconversion to PD or dementia with Lewy bodies in the same study. Moreover, in our study L5, representing activity levels during the least active 5 h, was elevated in iRBD patients, which has also been reported by Liguori et al. (2021) but not Feng et al. (2020) L5 elevation in iRBD could be related to more nighttime movements due to the loss of muscle atonia during REM sleep. The absence of such a finding by Feng et al. (2020) could be related to differences in the devices used (an Axivity triaxial accelerometer in the present study vs. a count-based Philips Actiwatch Spectrum Plus) or differences in the clinical patient sample.

Importantly, our results are also congruous with a study of rest-activity measures in healthy individuals who subsequently progressed to PD (Leng et al., 2020). Leng et al. (2020) found that lower amplitude and mesor were associated with a greater risk of progressing to PD. Given that 80% of individuals with iRBD progress to PD or dementia with Lewy bodies within 10 years (Galbiati et al., 2019; Postuma et al., 2019), our findings provide further evidence that sleep-wake activity is altered years before clinically manifest Lewy body disease, and could be used as predictive markers of disease progression.

Our study is to our knowledge the first to systematically describe fPCA characteristic activity patterns in 24-h cycles in iRBD. Besides the main finding of Component 1, which can be understood as a pattern of overall reduced daytime and increased nighttime activity in iRBD consistent with prior studies (Filardi et al., 2020; Liguori et al., 2021), Component 2 showed in iRBD both a delayed and low rise of activity levels during the morning hours, congruent with the delayed M10 start times we also observed in this study. Slowed activation in the morning hours was also suggested by a prior report of increased “sleep inertia” in patients with iRBD, i.e., increased time from wake to reaching average daytime motor activity (Liguori et al., 2021). Unlike other studies (Filardi et al., 2020; Liguori et al., 2021), given the large uncertainty in estimate napping behaviors, we did not formally include napping in our model. Napping may be another difference (Liguori et al., 2021) as well as a predictor of phenoconversion in iRBD (Feng et al., 2020), and may be reflected in differences in Component 1 and 3 in our data.

## Limitations

The cross-sectional nature of our iRBD patient data limits our analyses such that we cannot determine whether the severity of rest-activity measure alterations are predictive of subsequent Lewy body disease. Our analyses did not estimate daytime napping behavior or nighttime sleep. Time in bed information is not available in the UK Biobank accelerometer dataset so we could not accurately detect sleep in order to calculate sleep duration or sleep quality measures. Rather, our analyses focused on 24-h measures of rest-activity measures that reflect levels of activity but are agnostic to sleep-wake state. Finally, we may expect differences in habits and behaviors related to the geographic and temporal heterogeneity between cases and control participants included in this study. Actigraphy data in iRBD was collected in Northern California and 7–10 years later than the in the control participants of the UK Biobank. The study design did not allow to control for the influence weather (seasonality or geographical climate), culture, and personal habits in terms of walking vs. driving or use of public transports, physical exercise or other daily routines that could affect sleep and wake and activity levels over 24 h cycles.

## Future directions

Future research should extend these findings to longitudinal cohorts, and should capture a broader spectrum of individuals at risk for PD, DLB, or multiple system atrophy. Recent advancements in synuclein *in vivo* biomarkers (Okuzumi et al., 2023) could also be used to compare RAR in prodromal synuclein-positive individuals with and without RBD to better characterize synucleinopathy phenotype(s) associated with early RBD.

## Data availability statement

The raw data supporting the conclusions of this article will be made available by the authors, without undue reservation.

## Ethics statement

The studies involving humans were approved by the Institutional Review Board of Stanford and approval for the UK Biobank study was obtained from the North West Multicentre Research Ethics Committee, the National Information Governance Board for Health and Social Care in England and Wales, and the Community Health Index Advisory Group in Scotland. The studies were conducted in accordance with the local legislation and institutional requirements. The participants provided their written informed consent to participate in this study.

## Author contributions

JW: Conceptualization, Data curation, Formal analysis, Investigation, Methodology, Writing—original draft, Writing—review & editing. RL: Data curation, Investigation, Writing—review & editing. AE: Data curation, Investigation, Project administration, Writing—review & editing. FB: Data curation, Investigation, Project administration, Writing—review & editing. KP: Investigation, Writing—review & editing. JZ: Conceptualization, Investigation, Writing—review & editing. ED: Conceptualization, Investigation, Methodology, Project administration, Writing—original draft, Writing—review & editing.
